# Supplementary material for: Leishmania infection-derived extracellular vesicles drive transcription of genes involved in M2 polarization
Source: Front Cell Infect Microbiol. 2022 Aug 25;12:934611. doi: 10.3389/fcimb.2022.934611 (PMC9455154; doi:10.3389/fcimb.2022.934611)
Supplement: Supplementary Table 1 — Primers used in the study. [file Table_1.pdf]

Supplemental Table 1. Primers

Target	Forward 5' to 3'	Reverse 5' to 3'	M1/M2
iNOS	CAGCTGGGCTGTACAAACCTT	CATTGGAAGTGAAGCGGTTCG	M1
IFNγ	ACGCTTATGTTGTTGCTGATGG	CTTCCTCATGGCTGTTTCTGG	M1
TNFα	CGATGGGTTGTACCTTGTCTAC	GAGGTTGACTTTCTCCTGGTATG	M1
Arg-1	CAGAAGAATGGAAGAGTCAG	CAGATATGCAGGGAGTCACC	M2
IL-4R	CAGACCCGAAGCCAGGAGGAGTCAACC	GTTTGTGCAGGCAGTGAAGCAAGG G	M2
IL-10	GGCCCAGAAATCAAGGAGCA	AGACACCTTGGTCTTGGAGCTTAT	M2
GAPDH	ACTCCACTCACGGCAAATTC	CCAGTAGACTCCACGACATACT	Housekeeping
