# Supplementary figures and images for: Leishmania infection-derived extracellular vesicles drive transcription of genes involved in M2 polarization
Source: Front Cell Infect Microbiol. 2022 Aug 25;12:934611. doi: 10.3389/fcimb.2022.934611 (PMC9455154; doi:10.3389/fcimb.2022.934611)

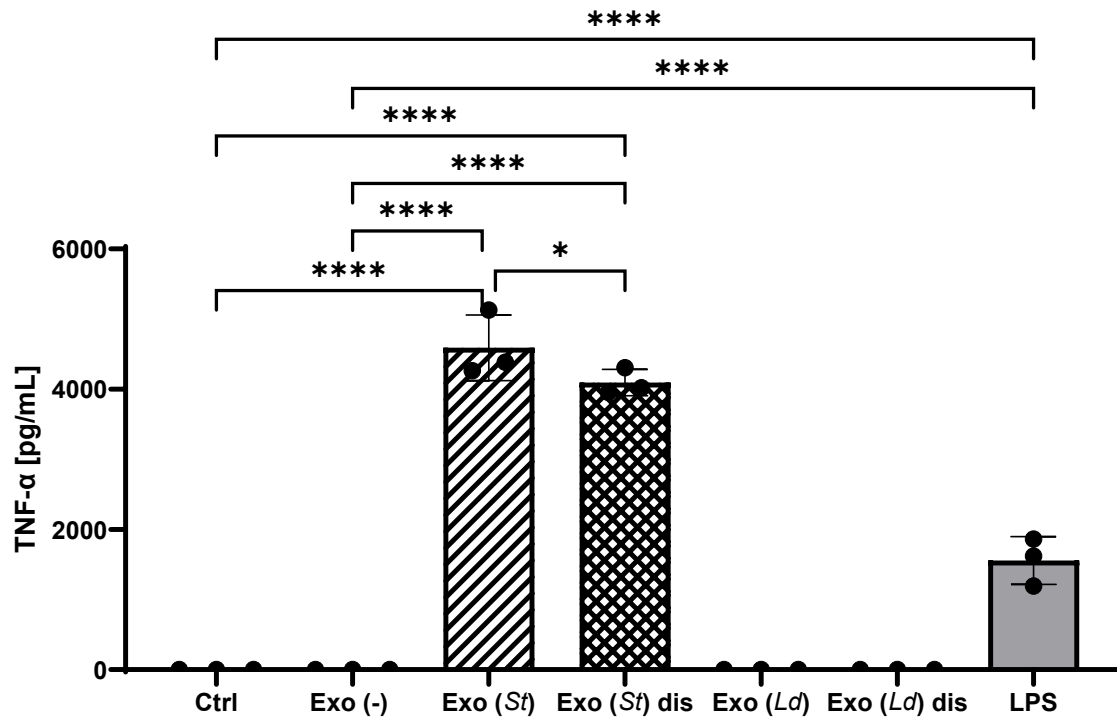

Supplement: Supplementary Figure 1 — The effect of EVs derived from L. donovani- or S. Typhimurium -infected macrophages on TNF-alpha release. RAW 264.7 macrophages were infected with L. donovani (72 hpi) or S. Typhimurium (MOI 5:1, 24 hpi). The EVs obtained from these cells were used to treat primary macrophages for 24 hours. Media (Ctrl) or EVs from uninfected cells were used as negative controls, and LPS treatment 100 ng/mL was used as a control treatment. The cell culture supernatants were collected and processed for anti-TNF-alpha ELISA. One-way ANOVA for multiple comparison tests was used to establish statistical significance. N=3. Exo (-), EVs from uninfected cells; Exo (Ld), EVs from cells infected with L. donovani; Exo (St), EVs from cells infected with S. Typhimurium; Exo (St) dis, disrupted EVs from cells infected with S. Typhimurium, Ctrl, cells not treated. [file Image_1.pdf]
